# Cigarette smoking and risk of non-Hodgkin lymphoma subtypes among women

**DOI:** 10.1038/sj.bjc.6601388

**Published:** 2003-11-25

**Authors:** L M Morton, T R Holford, B Leaderer, P Boyle, S H Zahm, Y Zhang, S Flynn, G Tallini, B Zhang, P H Owens, T Zheng

**Affiliations:** 1 Department of Epidemiology and Public Health, Yale University School of Medicine, 129 Church Street, Suite 700, New Haven, CT 06510, USA; 2 Department of Epidemiology and Biostatistics, European Institute of Oncology, Milan 20141, Italy; 3 Division of Cancer Epidemiology and Genetics, National Cancer Institute, NIH, DHHS, Bethesda, MD 20892, USA; 4 Department of Pathology, Yale University School of Medicine, New Haven, CT 06520, USA; 5 Department of Epidemiology and Biostatistics, McGill University, Montreal 3, Canada H3A1A2

**Keywords:** lymphoma, non-Hodgkin, smoking, women, case–control studies

## Abstract

Previous studies of the relationship between cigarette smoking and non-Hodgkin lymphoma (NHL) have yielded conflicting results, perhaps because most studies have evaluated the risk for all NHL subtypes combined. Data from a population-based case–control study conducted among women in Connecticut were used to evaluate the impact of cigarette smoking on the risk of NHL by histologic type, tumour grade, and immunologic type. A total of 601 histologically confirmed, incident cases of NHL and 718 population-based controls provided in-person interviews. A standardised, structured questionnaire was used to collect information on each subject's current smoking status, age at initiation, duration and intensity of smoking, and cumulative lifetime exposure to smoking. Our data suggest that cigarette smoking does not alter the risk of all NHL subtypes combined. However, increased risk of follicular lymphoma appears to be associated with increased intensity and duration of smoking, and cumulative lifetime exposure to smoking. Compared with nonsmokers, women with a cumulative lifetime exposure of 16–33 pack-years and 34 pack-years or greater experience 50% increased risk (OR=1.5, 95% CI 0.9–2.5) and 80% increased risk (OR=1.8, 95% CI 1.1–3.2), respectively, of follicular lymphoma (*P* for linear trend=0.05). Our study findings are consistent with several previous epidemiologic studies suggesting that cigarette smoking increases the risk of follicular lymphoma. This research highlights the importance of distinguishing between NHL subtypes in future research on the aetiology of NHL.

Over the past several decades, incidence rates of non-Hodgkin lymphoma (NHL) have increased dramatically in the United States and throughout the world (Hartge *et al*, 1994; Bray *et al*, 2001; Clarke and Glaser, 2002). The annual age-adjusted incidence rate of NHL in the US per 100 000 persons rose from 8.9 in 1973 to 15.6 in 1999 (Ries *et al*, 2002). Despite this observed increase in NHL incidence and the intensification of epidemiologic investigations into the aetiology of NHL, few concrete risk factors have been identified for NHL.

Cigarette smoking has been proposed as one factor that could potentially increase the risk of NHL, although epidemiologic investigations of smoking and NHL have yielded conflicting results (Magnani *et al*, 1990; Brown *et al*, 1992; Linet *et al*, 1992; Tavani *et al*, 1994; Nelson *et al*, 1997; Zahm *et al*, 1997; Adami *et al*, 1998; De Stefani *et al*, 1998; Freedman *et al*, 1998; Herrinton and Friedman, 1998; Holly *et al*, 1999; Miligi *et al*, 1999; Grant, 2000; Parker *et al*, 2000; Fabbro-Peray *et al*, 2001; Peach and Barnett, 2001; Stagnaro *et al*, 2001; Besson *et al*, 2003). Three studies have reported a statistically significant increased risk of NHL among smokers (Magnani *et al*, 1990; Linet *et al*, 1992; Freedman *et al*, 1998), while eight studies have reported no association or a weak association between cigarette smoking and NHL (Tavani *et al*, 1994; Nelson *et al*, 1997; Adami *et al*, 1998; De Stefani *et al*, 1998; Holly *et al*, 1999; Miligi *et al*, 1999; Grant, 2000; Fabbro-Peray *et al*, 2001).

A potential limitation of most previous studies of cigarette smoking and NHL may be that they have failed to investigate the effect of smoking by NHL subtype. Descriptive epidemiologic studies have shown that the magnitude of incidence rates and the recent changes in incidence rates vary for different NHL subtypes (Groves *et al*, 2000). Analytical epidemiologic studies have also shown that risk associated with various potential risk factors for NHL, such as pesticides, solvents, hair dye, and viruses, may vary by NHL subtype (Weisenburger, 1994; Germanidis *et al*, 1999; Shariff *et al*, 1999; Waddell *et al*, 2001; Zheng *et al*, 2002; Zhang *et al*, in press). Earlier epidemiologic studies of smoking and NHL that have considered the association between cigarette smoking and NHL have also suggested that the relationship may vary by histologic subtype of NHL (Herrinton and Friedman, 1998; Parker *et al*, 2000; Stagnaro *et al*, 2001; Besson *et al*, 2003). Thus, evaluation of the potential impact of cigarette smoking on NHL by disease subtype appears to be necessary.

Based on the inconsistent results of previous research, a population-based case–control study among 1319 Connecticut women was used to investigate the *a priori* hypothesis that smoking increases the risk of NHL, and that this increased risk varies by NHL histologic type, tumour grade, and immunologic type. The current status of smoking, age at initiation, duration and intensity of smoking, and cumulative lifetime exposure to smoking were compared between cases and controls to estimate the risk these factors confer on NHL and NHL subtypes.

## MATERIALS AND METHODS

### Study population

Detailed information on the study population and data collection for this study has been published elsewhere (Morton *et al*, in press). Briefly, a total of 1319 female residents of Connecticut, who were between the ages of 21 and 84 years, had no previous diagnosis of cancer (except nonmelanoma skin cancer), and were alive at the time of interview were recruited for this population-based case–control study from 1995 to 2001. The Yale Comprehensive Cancer Center's Rapid Case Ascertainment Shared Resource (RCA), a component of the Connecticut Tumor Registry (CTR), was used to identify incident cases of NHL (ICD-O, M-9590-9642, 9690-9701, 9740-9750). A total of 1122 potential NHL cases were identified, of which 290 were judged to be ineligible for this study (167 died before they could be interviewed, and 123 were excluded for other reasons, such as previous diagnosis of cancer, unable to speak English, or physician refusal). Thus, a total of 832 incident, eligible cases of NHL were contacted for participation in this study, and 601 (72%) of these cases completed in-person interviews. The median time between diagnosis and interview for cases was 2.5 months. Two study pathologists (Drs Flynn, Tallini) independently reviewed the tissue samples for all cases to confirm the diagnosis of NHL and classify NHL cases according to the Working Formulation into histologic type (diffuse, follicular, other), tumour grade (low, intermediate, high, other), and immunologic type (B-cell, T-cell, other). Tissue samples receiving conflicting classifications were re-evaluated until a consensus was reached.

A population-based control group, consisting of 718 women who completed in-person interviews, was assembled using two methods. Random digit dialing was used to recruit women less than 65 years of age; including the initial telephone screening, 69% of the women contacted using random digit dialing completed in-person interviews. Women 65 years of age and older were selected randomly from the files of the Centers for Medicare and Medicaid Services; 47% of the women contacted from these files completed in-person interviews. The number of controls that were randomly selected within each age stratum was adjusted every few months in order to frequency match cases and controls by age in 5-year groups. A total of 718 controls completed in-person interviews.

### Data collection

This study was conducted based on a protocol approved by the Human Investigations Committees at Yale University and the Connecticut Department of Health, and an Institutional Review Board of the National Cancer Institute. Cases were contacted first by letter and then by telephone, only after approval by each subject's physician. Controls were contacted after selection through random sampling. Trained interviewers administered a standardised, structured questionnaire to subjects who agreed to participate. During the interview, respondents were asked about their smoking history and other known or suspected risk factors for NHL.

Subjects were classified as never smokers if they had never smoked at least 100 cigarettes prior to 1 year before the interview. Respondents who had smoked at least 100 cigarettes were further questioned as to the age they began smoking, the average number of cigarettes smoked per day, the number of years during which they smoked at least one cigarette per day, whether they had stopped smoking in the past year, and the age at which they stopped smoking. Additional information on age, height, usual weight, education, race, alcohol use, menopausal status, family history of cancer, and other factors was also obtained during the interview. Continuous demographic variables and potential confounding factors were categorised *a priori*, based on previous cutpoints used in the literature or the distribution among control subjects.

### Statistical analysis

Statistical analyses for this study were performed using the SAS system, version 8.02. (SAS Institute Inc., Cary, NC, USA). Unconditional logistic regression models were developed using data on smoking history, in order to predict the risk of NHL and NHL subtypes (Breslow and Day, 1980). Subjects who reported smoking less than a total of 100 cigarettes in their lifetime were classified as never smokers, and were used as the reference group for all analyses. Ever smokers were further categorised as current or former smokers, based on their smoking status at the time of the interview. Data on the duration and intensity of smoking were integrated to create a single measure (pack-years=number of packs smoked per day times number of years smoked), in order to estimate the cumulative lifetime exposure to cigarette smoking. Continuous predictor variables, including age started smoking, duration of smoking, number of cigarettes smoked per day, and pack-years, were categorised into tertiles or quartiles *a priori*, based on previous cutpoints used in the literature or the distribution among control subjects.

Age, race, family history of cancer, education, body-mass index (BMI), alcohol use, and menopausal status were considered as potential confounding factors for this analysis. The associations of potential confounding factors with smoking predictor variables and NHL subtypes were assessed using Pearson's *χ*^2^ statistic. Wald *χ*^2^ statistics were calculated for each potential confounder in the multivariate models to test the null hypothesis that each covariate had no effect on the outcome, given that the other variables were already in the model. Decisions on which covariates to include in the final model were based on a Wald *χ*^2^ statistic with *P*<0.2 and a greater than 10% change in the risk estimates for at least some NHL subtypes. The final estimates of the risk of NHL and NHL subtypes using the smoking predictor variables were adjusted for age (<50, 50–70, >70 years), race (white, other), family history of cancer (any cancer, none), education (high school or less, some college, college graduate or more), BMI (<25, 25–29.99, ⩾30 kg m^−2^), alcohol use (ever, never), and menopausal status (premenopausal, postmenopausal). Adjusted odds ratios (OR) and 95% confidence intervals (95% CI) were calculated using multivariate unconditional logistic regression models. Tests for linear trend were conducted by including smoking predictor variables as continuous variables in the multivariate unconditional logistic regression models.

## RESULTS

The selected demographic characteristics of cases and controls were compared (Table 1

**Table 1:** Selected characteristics of NHL cases and controls among women from Connecticut

	**Percent of cases (*n*=601)**	**Percent of controls (*n*=718)**
*Age (years)*		
⩽45	12.3	15.9
46–55	19.3	18.9
56–65	20.6	19.9
66–75	28.6	25.9
⩾76	19.1	19.4
		
*Race*		
White	95.3	94.3
Other	4.7	5.7
		
*Family history*		
None	21.5	24.9
Any Cancer	78.5	75.1
		
*Education*		
High school or less	43.4	37.0
Some college	32.9	30.8
College graduate or more	23.6	32.2
		
*BMI* (*kg* *m*^−*2*^)		
<25	49.8	56.5
25–29.99	31.6	27.9
⩾30	18.6	15.6
		
*Alcohol use*		
Never	38.3	32.5
Ever	61.7	67.5

). Owing to the frequency matching by age strata, cases and controls were similar with respect to age. The distribution of race and reported family history of cancer were also similar among cases and controls. For all NHL cases combined and across most NHL subtypes (data not shown), cases tended to be less highly educated and have a higher BMI than controls (*P*<0.05). In addition, fewer cases than controls reported ever having consumed alcohol (*P*<0.05).

Table 2

**Table 2:** Risk of NHL associated with cigarette smoking for all NHL cases combined

	**Cases**	**Controls**		
	**#**	**#**	**OR^a^**	**95% CI**
*Smoking*				
Never	270	323	1.0	
Ever	331	395	1.0	(0.8, 1.3)
Current	77	106	0.9	(0.6, 1.2)
Former	254	289	1.1	(0.8, 1.4)
				
*Age at initiation (years)*				
⩽15	74	89	1.0	(0.7, 1.5)
16–19	163	187	1.1	(0.8, 1.4)
⩾20	94	119	1.0	(0.7, 1.3)
*P* for linear trend			0.97	
				
*Intensity (# cig/day)*				
1–10	73	100	1.0	(0.7, 1.3)
11–15	83	104	1.0	(0.7, 1.4)
16–20	119	135	1.0	(0.8, 1.4)
>20	56	56	1.2	(0.8, 1.7)
*P* for linear trend			0.32	
				
*Duration (years)*				
1–10	50	72	0.9	(0.6, 1.4)
11–20	62	90	0.9	(0.6, 1.3)
21–30	69	80	1.0	(0.7, 1.5)
>30	150	153	1.2	(0.9, 1.5)
*P* for linear trend			0.45	
				
*Pack-years*				
1–7	81	103	1.0	(0.7, 1.5)
8–14	53	95	0.7	(0.5, 1.0)
15–33	94	104	1.1	(0.8, 1.5)
⩾34	103	93	1.3	(0.9, 1.8)
*P* for linear trend			0.18	

a Adjusted for age, race, family history of any cancer, education, BMI, previous alcohol use, and menopausal status.

presents the risk of NHL by smoking for all NHL subtypes combined. When compared to women who have never smoked, the risk of NHL did not appear to be significantly altered by current or former smoking status, the age subjects began smoking, the number of cigarettes smoked per day, the duration of smoking, or the cumulative lifetime exposure to smoking (Table 2).

Further analysis by NHL subtype, however, suggests that the risk of follicular lymphoma may be elevated by increased duration of smoking and cumulative lifetime exposure to smoking (Table 3

**Table 3:** Risk of NHL associated with cigarette smoking for NHL subtypes

	**Histologic type**				**Tumor grade**						**Immunologic type**			
	**Diffuse (*n*=346)**		**Follicular (*n*=134)**		**Low (*n*=177)**		**Intermediate (*n*=286)**		**High (*n*=18)**		**B cell (*n*=473)**		**T cell (*n*=44)**	
	**OR^a^**	**95% CI**	**OR^a^**	**95% CI**	**OR^a^**	**95% CI**	**OR^a^**	**95% CI**	**OR^a^**	**95% CI**	**OR^a^**	**95% CI**	**OR^a^**	**95% CI**
*Smoking*														
Never	1.0		1.0		1.0		1.0		1.0		1.0		1.0	
Ever	1.0	(0.7, 1.3)	1.2	(0.8, 1.8)	1.1	(0.8, 1.6)	1.0	(0.7, 1.3)	1.0	(0.4, 2.7)	1.0	(0.8, 1.3)	1.2	(0.6, 2.3)
Current	0.7	(0.4, 1.1)	1.6	(0.9, 2.8)	1.1	(0.6, 1.9)	0.8	(0.5, 1.3)	1.1	(0.2, 6.3)	0.9	(0.6, 1.3)	0.9	(0.3, 2.5)
Former	1.1	(0.8, 1.4)	1.2	(0.8, 1.8)	1.1	(0.8, 1.6)	1.1	(0.8, 1.5)	0.9	(0.3, 2.8)	1.1	(0.8, 1.4)	1.3	(0.6, 2.5)
														
*Age at initiation (years)*														
⩽15	1.1	(0.6, 1.6)	1.1	(0.6, 2.0)	0.8	(0.4, 1.5)	1.2	(0.8, 1.9)	1.8	(0.5, 6.6)	1.0	(0.7, 1.4)	1.6	(0.7, 4.1)
16–19	0.9	(0.7, 1.3)	1.5	(0.9, 2.4)	1.3	(0.9, 2.0)	1.0	(0.7, 1.4)	0.7	(0.2, 2.8)	1.1	(0.8, 1.5)	0.9	(0.4, 2.1)
⩾20	0.9	(0.6, 1.4)	1.0	(0.6, 1.8)	1.1	(0.6, 1.7)	0.9	(0.6, 1.4)	0.9	(0.2, 4.2)	1.0	(0.7, 1.4)	1.3	(0.6, 3.2)
*P* for linear trend	0.50		0.31		0.60		0.67		0.75		0.98		0.92	
														
*Intensity (#cig/day)*														
1–10	1.0	(0.7, 1.6)	0.6	(0.3, 1.3)	0.9	(0.5, 1.6)	0.9	(0.6, 1.5)	0.4	(0.1, 3.8)	0.9	(0.6, 1.3)	2.0	(0.7, 4.1)
11–15	0.9	(0.6, 1.3)	1.4	(0.8, 2.4)	1.2	(0.7, 1.9)	0.9	(0.6, 1.4)	1.5	(0.4, 6.1)	1.0	(0.7, 1.4)	0.9	(0.3, 2.6)
16–20	0.9	(0.7, 1.4)	1.7	(1.1, 2.7)	1.3	(0.9, 2.1)	1.0	(0.7, 1.5)	0.9	(0.2, 3.5)	1.2	(0.8, 1.6)	0.5	(0.2, 1.6)
>20	1.1	(0.7, 1.8)	0.9	(0.4, 2.0)	0.9	(0.4, 1.7)	1.2	(0.7, 1.9)	1.5	(0.3, 7.7)	1.0	(0.6, 1.6)	2.4	(1.0, 5.9)
*P* for linear trend	0.65		0.16		0.34		0.52		0.51		0.43		0.44	
														
*Duration (years)*														
1–10	1.1	(0.7, 1.8)	0.8	(0.4, 1.7)	0.8	(0.4, 1.5)	1.2	(0.7, 1.9)	1.0	(0.2, 5.2)	0.9	(0.6, 1.5)	0.8	(0.2, 2.8)
11–20	0.8	(0.5, 1.3)	0.9	(0.5, 1.8)	0.9	(0.5, 1.6)	0.8	(0.5, 1.4)	0.4	(0.1, 3.7)	0.9	(0.6, 1.4)	0.6	(0.2, 2.0)
21–30	0.9	(0.6, 1.4)	1.1	(0.6, 2.0)	1.1	(0.6, 1.9)	0.8	(0.5, 1.3)	0.9	(0.2, 4.6)	1.0	(0.6, 1.4)	1.6	(0.6, 4.0)
>30	1.0	(0.7, 1.4)	1.8	(1.1, 2.8)	1.4	(0.9, 2.1)	1.1	(0.8, 1.6)	1.6	(0.4, 5.9)	1.2	(0.8, 1.6)	1.6	(0.7, 3.7)
*P* for linear trend	0.91		0.06		0.27		0.78		0.56		0.55		0.32	
														
*Pack-years*														
1–7	1.1	(0.7, 1.7)	0.9	(0.5, 1.7)	1.0	(0.6, 1.7)	1.1	(0.7, 1.7)	0.8	(0.2, 3.8)	1.1	(0.8, 1.6)	0.8	(0.3, 2.4)
8–14	0.6	(0.4, 1.0)	0.8	(0.4, 1.5)	0.9	(0.5, 1.5)	0.6	(0.3, 1.0)	0.4	(0.1, 3.7)	0.7	(0.4, 1.0)	1.2	(0.4, 3.1)
15–33	1.0	(0.7, 1.5)	1.5	(0.9, 2.5)	1.2	(0.7, 2.0)	1.1	(0.7, 1.6)	1.8	(0.5, 6.2)	1.1	(0.7, 1.5)	1.6	(0.7, 3.7)
⩾34	1.1	(0.7, 1.6)	1.8	(1.1, 3.2)	1.4	(0.9, 2.3)	1.3	(0.8, 1.9)	1.1	(0.2, 5.6)	1.3	(0.9, 1.8)	1.2	(0.5, 3.3)
*P* for linear trend	0.66		0.05		0.28		0.44		0.09		0.31		0.31	

a Adjusted for age, race, family history of any cancer, education, BMI, previous alcohol use, and menopausal status.

). Compared with nonsmokers, women with a cumulative lifetime exposure of 16–33 pack-years and 34 pack-years or greater experience 50% increased risk (OR=1.5, 95% CI 0.9–2.5) and 80% increased risk (OR=1.8, 95% CI 1.1–3.2), respectively, illustrating a dose–response relationship between cumulative lifetime exposure and follicular lymphoma (*P* for linear trend=0.05). Our data fail to show clear relationships with the intensity of smoking, the age subjects began smoking, and current smoking status, although this is not surprising given the small number of cases examined if the relationship with smoking is weak. Stratification by age for those aged less than 65 years or 65 years and over showed similar results.

Smoking history does not appear to alter the risk of NHL by tumour grade (low, medium, high) or for B-cell lymphomas (Table 3). There is a suggestion of an association for T-cell lymphomas; however, the number of cases was too small to conclusively assess the impact of smoking. Smoking did not significantly alter the risk of NHL for other histologic types, tumour grades, and immunologic types, as classified by the Working Formulation (data not shown).

## DISCUSSION

In this population-based case–control study, we found that smoking appeared to increase the risk of follicular lymphoma, and that this risk increased with increased duration and pack-years of cigarette smoking. Smoking did not appear to play a role in other histologic types of NHL.

Our results are consistent with previous epidemiologic studies that also considered the relationship between cigarette smoking and NHL by histologic subtype, and that found an increased risk among smokers for follicular lymphoma, but not for diffuse lymphoma (Herrinton and Friedman, 1998; Parker *et al*, 2000; Stagnaro *et al*, 2001; Besson *et al*, 2003). In a population-based case–control study conducted in Italy, smoking was associated with increased risk of follicular lymphoma, particularly among women (Stagnaro *et al*, 2001). That study reported approximately two-fold increased risk among women with the longest duration of smoking; however, the trend for intensity of smoking was less clear, and the impact of cumulative lifetime exposure to cigarette smoking was not evaluated. A hospital-based case–control study among men and women in France suggested a three-fold increase in the risk of follicular NHL among current smokers, although the result was not statistically significant, perhaps due to the small sample size (Besson *et al*, 2003). Two cohort studies, the Iowa Women's Health Study (Parker *et al*, 2000) and a cohort study of men and women from California (Herrinton and Friedman, 1998), also have reported approximately two-fold increased risk of follicular lymphoma among smokers compared to nonsmokers, although these estimates were based on very few cases. Our study and the three studies discussed above all found that a history of cigarette smoking is associated with an approximately two-fold or greater increased risk of follicular lymphoma when compared to nonsmokers, but no relationship was found between smoking and other NHL subtypes.

Previous studies that did not investigate the relationship between smoking and NHL by histologic subtype may not have found a relationship because smoking seems to increase only the risk of follicular lymphoma, but not other NHL subtypes. If we had not stratified by histologic type, the results of this study would have been consistent with previous studies that did not stratify and showed no effect.

It is not clear why the relationship between smoking and NHL only exists for follicular lymphoma and not for other subtypes of NHL. The mechanism of follicular lymphoma development among cigarette smokers is not known. Studies have shown that approximately 80–90% of follicular lymphomas, regardless of cell type, are positive for t(14;18) translocation (Cleary *et al*, 1986a,Cleary *et al*, 1986b). This somatic mutation joins the bcl-2 gene on chromosome 18 to the immunoglobulin heavy chain gene on chromosome 14, increasing the production of bcl-2 protein, which is involved in the inhibition of apoptosis (Cleary *et al*, 1986a,Cleary *et al*, 1986b). Since these mutations are likely to be the result of a direct carcinogenic effect, rather than being related to immune suppression, it is possible that the carcinogenic compounds in cigarettes could play a role in these mutations. A recent case–control study of NHL that classified cases by this mutation suggested a potential increased risk among cigarette smokers for t(14;18)-positive NHL but not for t(14;18)-negative NHL, although no clear trend was observed (Schroeder *et al*, 2002). Additional studies considering the role of this mutation could help to elucidate the role of cigarette smoking in the development of follicular lymphomas.

A number of strengths and limitations of this study should be considered in the interpretation of the results. In this large, population-based case–control study, trained interviewers used in-person, standardised, and structured interviews to minimise the information bias resulting from exposure misclassification. Incident cases were histologically confirmed and categorised according to the Working Formulation, in order to minimise the information bias resulting from disease misclassification. In addition, subjects were asked standard, detailed questions with respect to their smoking history, so that the smoking status, the age subjects began smoking, the intensity and duration of smoking, and the lifetime cumulative exposure to cigarette smoke could be evaluated separately to understand which aspect of cigarette smoking, if any, affects the risk of NHL and NHL subtypes. Despite the relatively large size of this study, however, stratification limited our ability to estimate the impact of smoking on the risk of high-grade tumours and T-cell lymphomas, which account for only a small proportion of lymphomas. In addition, this analysis only addresses the potential relationship between smoking and NHL for women, because one of the main objectives of this population-based case–control study was to investigate the relationship between hair dye use, female reproductive history, and menarche and risk of NHL in Connecticut women.

Although this study was population-based, the relatively low participation rates among potential population controls, especially among women 65 years of age and older, recruited from the files of the Centers of Medicare and Medicaid Services, are of possible concern. However, the association between smoking and disease was observed only among women with follicular lymphomas and not other NHL subtypes. In addition, stratification by age for those aged less than 65 years or 65 years and over showed similar results. Therefore, it is unlikely that selection bias can explain the observed associations. Self-report of smoking history has been shown to be reliable and accurate, based on comparisons of self-reported data to biochemical markers of tobacco exposure (Patrick *et al*, 1994). Therefore, it is unlikely that the observed result is due to recall bias. The hypothesised relationship between smoking and NHL is not well known; therefore it is unlikely that the observed result is due to reporting bias. In addition, the elevated risk of follicular lymphoma is unlikely to be the result of recall or reporting bias because the association is not observed across all NHL subtypes.

In summary, this population-based case–control study supports the hypothesis that increased pack-years and duration of smoking result in an elevated risk of follicular lymphoma, but do not alter the risk for other NHL subtypes. The results of this study highlight the need to distinguish between NHL histologic subtypes in future research on the aetiology of NHL.
